# Shufeiya Recipe Improves Monocrotaline-Induced Pulmonary Hypertension in Rats by Regulating SIRT3/FOXO3a and Its Downstream Signaling Pathways

**DOI:** 10.1155/2022/3229888

**Published:** 2022-02-18

**Authors:** Zhuangzhuang Jia, Haifeng Yan, Shuai Wang, Lin Wang, Yawen Cao, Shanshan Lin, Zeyu Zhang, Ci Wang, Xianliang Wang, Jingyuan Mao

**Affiliations:** ^1^Department of Cardiovascular Diseases, First Teaching Hospital of Tianjin University of Traditional Chinese Medicine, National Clinical Research Center for Chinese Medicine Acupuncture and Moxibustion, Tianjin 300381, China; ^2^Tianjin University of Traditional Chinese Medicine, Tianjin 301617, China; ^3^Department of Cardiovascular Diseases, The First Affiliated Hospital of Henan University of Traditional Chinese Medicine, Zhengzhou, Henan 450000, China

## Abstract

Pulmonary hypertension (PH) is a chronic and progressive disease caused by obstructions and functional changes of small pulmonary arteries. Current treatment options of PH are costly with patients needing long-term taking medicine. The traditional Chinese medicine (TCM) compound “Shufeiya Recipe” was used to intervene in monocrotaline- (MCT-) induced pulmonary hypertension in rats. The rats were randomly divided into the control group, model group, positive drug (Sildenafil) group, and Shufeiya Recipe low-, moderate-, and high-dose groups. The improvement effect of the Shufeiya Recipe on the mean pulmonary artery pressure (mPAP) was assessed in PH rats, and pathological staining was used to observe the pathological changes of lung tissue. The impact of the Shufeiya Recipe on oxidative stress damage in rats with pulmonary hypertension and the regulation of SIRT3/FOXO3a and its downstream signaling pathways were determined. The results showed that Shufeiya Recipe could significantly downregulate mPAP and improve lung histopathological changes; downregulate serum levels of reactive oxygen species (ROS); upregulate the concentrations of COX-1 and COX-2 and the activity of Mn-SOD; inhibit oxidative response damage; promote the protein expression of SIRT3, FOXO3a, p-PI3K, p-AKT, and p-eNOS; increase the level of expression of NO, sGC, cGMP, and PKG; and downregulate the level of protein expression of Ras, p-MEK1/2, p-ERK1/2 and c-fos. These results indicate that Shufeiya Recipe can improve MCT-induced pulmonary hypertension in rats by regulating SIRT3/FOXO3a and its downstream PI3K/AKT/eNOS and Ras/ERK signaling pathways.

## 1. Introduction

Pulmonary hypertension (PH) is a chronic and progressive disease caused by obstruction of small pulmonary arteries. It is characterized by pulmonary vascular remodeling, increased pulmonary circulation resistance, and elevated pulmonary artery pressure (with an average pulmonary artery pressure at rest of ≥25 mmHg), ultimately leading to right heart failure and even death [1]. As many as 1% of patients are affected by pulmonary hypertension globally, with a prevalence of around 10% for people over 65 years old [2]. It is estimated that the 1-year mortality rate of patients with pulmonary hypertension is about 15%, while the 3-years mortality rate is approximately 30% [3]. Currently, most therapeutic drugs work by relaxing the blood vessels and inhibiting the proliferation of pulmonary artery smooth muscle cells [4]. These drugs are quite expensive, and PH patients often require intensive clinical care [5]. Therefore, it is of great significance to seek more effective prevention and control measures.

The occurrence and development of pulmonary hypertension involve many factors, such as hypoxia, inflammation, oxidative stress, and endothelial dysfunction, to name a few [6, 7]. Among them, oxidative stress is an important precursor to PH development [8]. An imbalance between the generation and removal of oxygen free radicals in the body and the excessive accumulation of ROS could promote the proliferation, vasoconstriction, and reconstruction of smooth muscle cells [9]. Mitochondria are the primary site of ROS generation. When mitochondrial dysfunction occurs, this can lead to oxidative stress damage [10, 11]. Under normal circumstances, cells can inhibit the overproduction of ROS and maintain its normal function by increasing the activity of antioxidant enzymes, such as catalase (CAT), manganese superoxide dismutase (Mn-SOD), and glutathione peroxidase (GSH-Px). [12]. Previous studies have shown that silent information regulator3 (SIRT3) could increase the expression level of forkhead box O3 (FOXO3a) and enhance the activity of MnSOD [13, 14]. The increase in the activity of Mn-SOD and CAT could reduce ROS generation, inhibiting the expression of Ras protein and promoting the activation of the PI3K/AKT signaling pathway. On the other hand, excessive ROS could activate the Ras protein, promoting the expression of downstream substrates MEK1/2 and ERK1 protein, which in turn mediate the increase in c-fos promoter activity, inducing cell proliferation and differentiation, and facilitating pulmonary hypertension [15, 16]. However, the activated upstream PI3K could trigger AKT, activating the phosphorylation sites of eNOS Ser1177 and Thr495 to promote NO synthesis [17–19]. Subsequently, the soluble guanylate cyclase (sGC) in the vascular smooth muscle cells could be activated, converting guanosine triphosphate (GTP) into cyclic guanosine monophosphate (cGMP), thereby activating cGMP-dependent protein kinases G (PKG), leading to vasodilation and limiting pulmonary hypertension [20–22].

Traditional Chinese medicine has the advantages of being a multi-component, multi-target, and multi-mechanistic treatment modality, which is gradually gaining recognition in the clinical treatment of PH. The traditional Chinese medicine compound “Shufeiya Recipe” comprises four Chinese herbs: *Radix Salviae*, *Carthami Flos*, *Cornus Officinalis Sieb. Et Zucc.*, and *Platycodon Grandiforus*. *Radix Salviae* and *Carthami Flos* were shown to promote blood circulation while inhibiting blood stasis, inflammation and oxidation [23, 24]. *Cornus Officinalis Sieb. Et Zucc.* was shown to protect the heart and the brain and act as an antioxidant as well as an immune regulator, etc. [25], while *Platycodon Grandiforus* could regulate pulmonary inflammation, inhibit cell apoptosis, and protect the heart [26–28]. In this study, Shufeiya Recipe was used as an intervention in MCT-induced pulmonary hypertension in rats to observe its effect in improving PH and regulating SIRT3/FOXO3a and its downstream PI3K/AKT/eNOS and Ras/ERK signaling pathways.

## 2. Materials and Methods

### 2.1. Animal

Specific pathogen-free (SPF) adult male Sprague-Dawley (SD) rats weighing 230~250 g were purchased from SiPeiFu Laboratory Animal Technology (Beijing, China) with laboratory animal license number: SCXK (Beijing) 2019-0010. All rats were housed in an air-conditioned room at 22 ± 2°C with a 12/12 hour light/dark cycle, in which the rats had free access to food and water.

### 2.2. Experimental Drugs

Shufeiya Recipe is composed of 20 g *Platycodon Grandiforus*, 15 g *Radix Salviae*, 15 g *Carthami Flos*, and 15 g *Cornus Officinalis Sieb. Et Zucc.* According to the human to rat dose conversion method, the dosage was adjusted to a low dose (crude drug concentration of 0.2925 g/ml), moderate dose (crude drug concentration of 0.5850 g/ml), and high dose (crude drug concentration of 1.1700 g/ml). The positive control drug used was sildenafil (Yuanye Bio-Technology Co., Ltd., China), the specification is 1 g, the batch number is X23A8Y42189, and a concentration of 2.5 mg/ml was prepared by dissolving in distilled water.

### 2.3. Modeling and Grouping Methods

60 SD rats were randomly divided into 6 groups: control group, model group, sildenafil group, and Shufeiya Recipe low- (SFY-L), moderate- (SFY-M), and high-dose (SFY-H) groups, with 10 rats in each group. A single intraperitoneal injection of MCT (60 mg/kg) was used to establish the PH rat model. The rats in the control group and the model group were given 1 ml/100 g distilled water by gavage, the sildenafil group was given sildenafil solution 25 mg/kg by gavage, and the low-, moderate-, and high-dose groups of Shufeiya Recipe were given a concentration of 0.2925 g/ml, 0.5850 g/ml, an d1.1700 g/ml of Shufeiya Recipe solution 1 ml/100 g by gavage, respectively, and each intervention was given by intragastric administration once daily for 14 consecutive days.

### 2.4. Determination of mPAP in Rats with Pulmonary Hypertension

After 14 days of the administration, the rats were anesthetized with 1.5% tribromoethanol (0.8 ml/100 g). Stripping the external jugular vein and ligating the distal end, then a 2 ~ 3 mm V-shaped opening was made at the proximal end of the external jugular vein. Next, a catheter was inserted into the V-shaped opening and was gently advanced. The changes of mPAP in rats were determined according to the waveform shown by the Hemodynamic Recording System.

### 2.5. Morphological Changes of Lung Tissues

The animals were anesthetized by intraperitoneal injection of tribromoethanol solution and the middle and upper lobes of the left lung of the rats were taken, rinsed with physiological saline, and fixed in 4% paraformaldehyde for 72 h. The specimens were made into paraffin-embedded sections with a thickness of 5 *μ*m along the hilum, and the slices were stained with conventional HE and mounted with neutral gum. The pathomorphological changes of the lung tissues of the rats in each group were observed under a microscope.

### 2.6. Detection of Oxidative Stress Levels

Enzyme-linked immunosorbent assay (ELISA) was used to detect ROS (JL21051, Jianglaibio Co. Ltd., China), cyclooxygenase-1 (COX-1) (RA21050, Bioswamp Co. Ltd., China), and cyclooxygenase-2 (COX-2) (RA20086, Bioswamp Co. Ltd., China) in the serum of rats with pulmonary hypertension. The hydroxylamine method was used to detect manganese superoxide dismutase (Mn-SOD) (A001-2, Jiancheng Bioengineering Institute, China). The specific steps were carried out in accordance with the manufacturer's requirements.

### 2.7. Detection of the Expression Levels of NO, sGC, and cGMP

The middle and upper lobe tissues of the left lung of rats were taken for homogenization. The microwell plate method was used to evaluate the nitric oxide (NO) content (JL13431, Jianglaibio Co. Ltd., China), and the ELISA was used to detect the soluble guanylyl cyclase (sGC) (JL46177, Jianglaibio Co. Ltd., China) content and cyclic guanosine monophosphate (cGMP) (JL11179, Jiangglaibio Co. Ltd., China) content. The specific steps were carried out in accordance with the manufacturer's requirements.

### 2.8. Western Blotting

The total protein of rat lung tissues in each group was extracted, and the protein concentration was measured using the bicinchoninic acid (BCA) method. The extracted proteins were then separated by sodium dodecyl sulfate-polyacrylamide gel electrophoresis (SDS-PAGE), and the proteins were then transferred onto the polyvinylidene fluoride (PVDF) membrane. After blocking with 5% skimmed milk powder for 2 h, primary antibodies SIRT3 (ab246522, 1 : 1000), FOXO3a (ab109629, 1 : 1000), p-PI3K (AF3242, 1 : 1000), AKT (CSB-PA008118, 1 : 1000), p-eNOS (CSB-PA000659, 1 : 1000), PKG (DF7018, 1 : 1000), Ras (ab52939, 1: 2000), p-MEK1/2 (AF8035, 1 : 1000), p-ERK1/2 (ab201015, 1 : 1000), and c-fos (ab134122, 1 : 2000) were added separately and incubated at 4°C overnight. The next day, after 1 h incubation with HRP-labeled secondary antibodies at room temperature, the target bands were exposed with an ultrasensitive multifunction imager, and the expression intensity of each target protein/*β*-actin was analyzed using ImageJ software 1.8.0.

### 2.9. Immunofluorescence Analysis

Paraffin sections of the lung tissue of rats were prepared. After deparaffinization and hydration, these sections were immersed in boiling sodium citrate solution for antigen retrieval and then blocked with normal goat serum for 30 min. The primary antibodies p-PI3K (AF3242, 1 : 1000), p-eNOS (CSB-PA000659, 1 : 200), and p-ERK1/2 (ab201015, 1 : 200) were added on the sections separately, which were placed in a humid box and incubated overnight at 4°C. The next day, FITC-labeled fluorescent secondary antibodies were incubated at 37°C in the dark. After staining the nucleus with DAPI solution, the high-power field of view was randomly selected and photographed using an Olympus inverted fluorescence microscope, and the statistical analysis was performed.

### 2.10. Statistical Analysis

All data were expressed as the mean ± standard deviation. The intergroup differences in the data were evaluated by one-way analysis of variance (ANOVA) with the least significant difference (LSD) or Tamhane's T2 post hoc analysis using SPSS 20.0. *P* < 0.05 was considered statistically significant.

## 3. Results

### 3.1. Effects on mPAP and Pathological Changes of Lung Tissue

**Figure 1:**
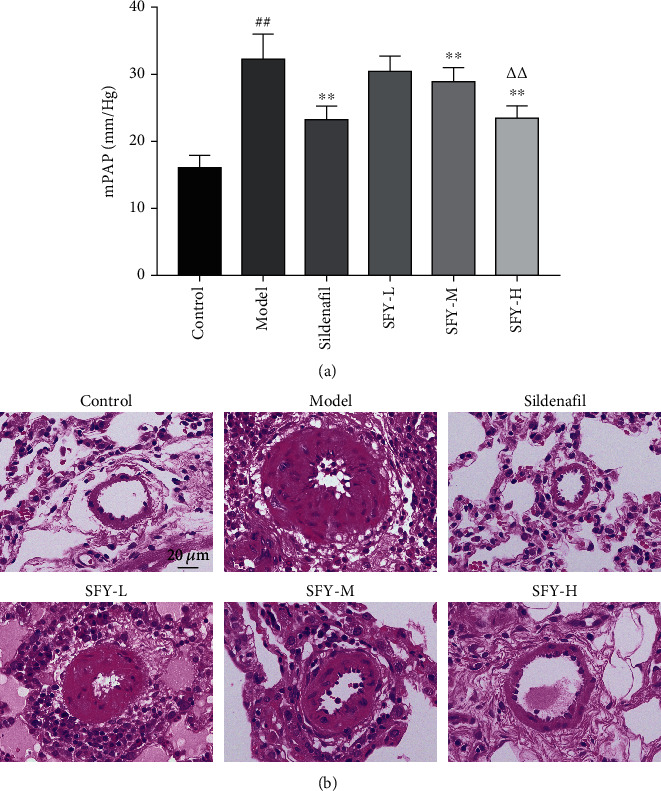
Shufeiya Recipe significantly relieved mPAP and pathological changes of lung tissue of PH rats. (a) Moderate and high doses of Shufeiya Recipe significantly decreased mPAP of PH rats. (b) Shufeiya Recipe relieved pathological changes of lung tissue of PH rats. *n* = 10; ^##^*P* < 0.01 vs. the control group, ^∗∗^*P* < 0.01 vs. the model group, and ^△△^*P* < 0.01 vs. the moderate-dose group of Shufeiya Recipe.

Compared with the control group, mPAP in the model group was significantly increased (*P* < 0.01). In contrast with the model group, mPAP in the sildenafil group and moderate- and high-dose groups of Shufeiya Recipe were significantly decreased (*P* < 0.01). The pulmonary tissue structure of rats in the control group was normal, showing intact, smooth, and neatly arranged intima of the pulmonary artery, and smooth muscle was not thickened. However, in the model group, the continuity of the pulmonary artery endothelium was damaged with irregular, intermittent hypertrophy of pulmonary arterioles, partially narrowed lumen, and significantly reduced effective area. In contrast with the model group, the pulmonary artery wall thickness and lumen stenosis of the sildenafil group and the Shufeiya Recipe groups showed significant improvement. Furthermore, the most marked improvement was obtained by the high-dose treatment of Shufeiya Recipe (Figure 1).

### 3.2. Effects on Oxidative Stress Levels

**Figure 2:**
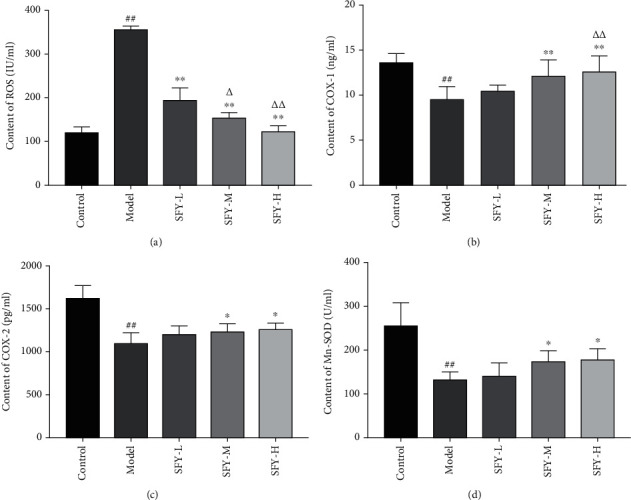
Shufeiya Recipe significantly inhibited the oxidative stress level of PH rats. (a) Shufeiya Recipe significantly downregulated the level of ROS accumulation of PH rats, *n* = 3. (b–d) Shufeiya Recipe increased COX-1, COX-2, and Mn-SOD contents of PH rats, *n* = 6. ^##^*P* < 0.01 vs. the control group; ^∗∗^*P* < 0.01 vs. the model group; and ^△^*P* < 0.05, ^△△^*P* < 0.01 vs. the low-dose group of Shufeiya Recipe.

Compared with the control group, the serum ROS content of the model group was significantly increased (*P* < 0.01), while the COX-1, COX-2, and Mn-SOD contents were significantly decreased (*P* < 0.01). As opposed to the model group, after the intervention of the high and moderate doses of Shufeiya Recipe, the oxidative stress level of PH rats was significantly inhibited, while the COX-1, COX-2, and Mn-SOD contents were significantly increased (*P* < 0.05, *P* < 0.01), and the level of ROS accumulation was significantly downregulated under each dose of Shufeiya Recipe treatment (*P* < 0.01) (Figure 2).

### 3.3. Effects on the SIRT3/FOXO3a Signaling Pathway

**Figure 3:**
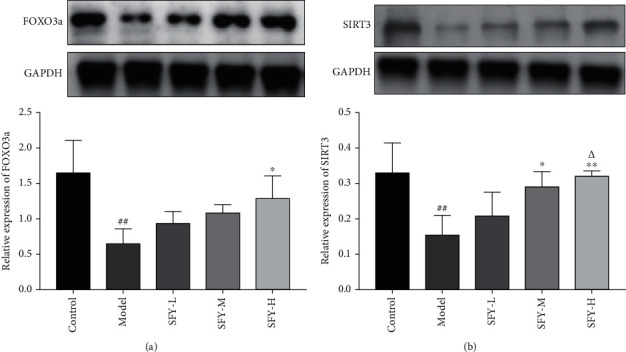
Shufeiya Recipe activated the SIRT3/FOXO3a signaling pathway of PH rats. (a) The high-dose group of Shufeiya Recipe stimulated the expression of FOXO3a. (b) The moderate- and high-dose groups of Shufeiya Recipe upregulated the expression level of SIRT3, and the most marked improvement was obtained by the high-dose treatment of Shufeiya Recipe. *n* = 3; ^##^*P* < 0.01 vs. the control group; ^∗^*P* < 0.05, ^∗∗^*P* < 0.01 vs. the model group; and ^△^*P* < 0.05 vs. the low-dose group of Shufeiya Recipe.

Contrasting with the control group, the protein expression level of SIRT3 and FOXO3a in the model group was visibly reduced (*P* < 0.01). On the other hand, compared with the model group, the expression level of SIRT3 protein in the Shufeiya Recipe high-dose group was dramatically increased (*P* < 0.05). In addition, the expression level of SIRT3 protein in the Shufeiya Recipe moderate-dose group and that of FOXO3a protein in the Shufeiya Recipe high-dose group were distinctively improved (*P* < 0.05) (Figure 3).

### 3.4. Effects on the PI3K/AKT/eNOS Signaling Pathway

**Figure 4:**
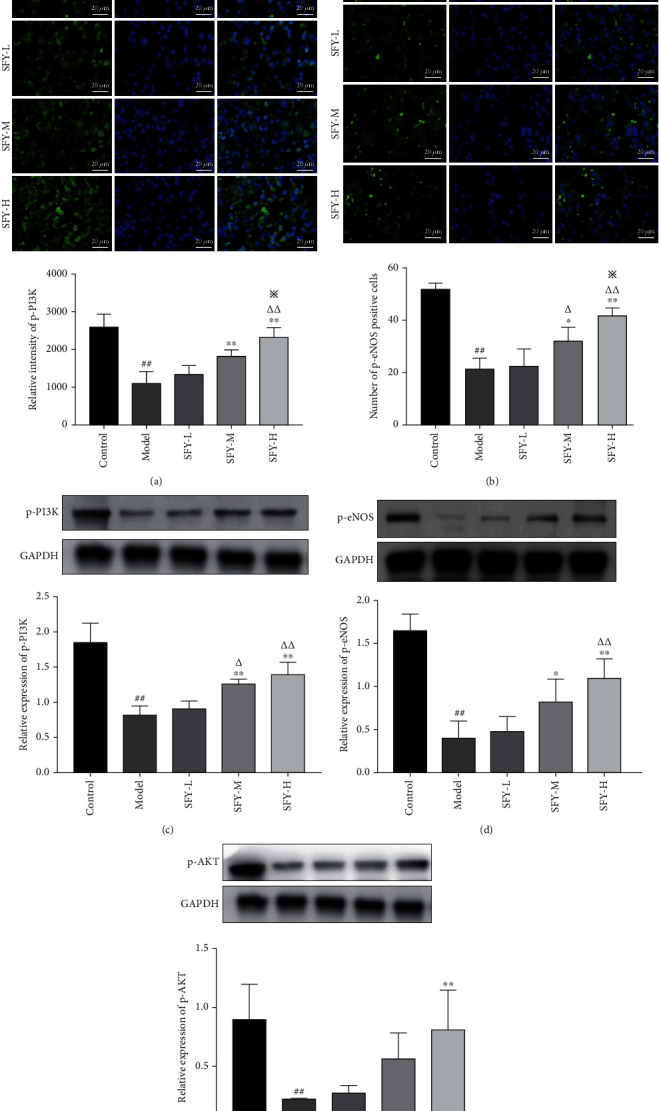
Shufeiya Recipe activated the PI3K/AKT/eNOS signaling pathway of PH rats. (a, b) Moderate and high groups of Shufeiya Recipe upregulated the relative immunofluorescence intensity of p-PI3K and p-eNOS. (c–e) Different doses of Shufeiya Recipe upregulated the protein expression level of p-PI3K, p-AKT, and p-eNOS. *n* = 3, ^##^*P* < 0.01 vs. the control group; ^∗^*P* < 0.05, ^∗∗^*P* < 0.01 vs. the model group; ^△^*P* < 0.05, ^△△^*P* < 0.01 vs. the low-dose group of Shufeiya Recipe; ^※^*P* < 0.05 vs. the moderate-dose group of Shufeiya Recipe.

The expression levels of p-PI3K, p-AKT, and p-eNOS in the model group were significantly reduced compared with the control group (*P* < 0.01). As opposed to the model group, the protein expression levels of p-PI3K in the moderate- and high-dose groups of Shufeiya Recipe were significantly increased (*P* < 0.01). Moreover, a high dose of Shufeiya Recipe also accelerated the expression of p-AKT(*P* < 0.01). Furthermore, the protein expression levels of p-eNOS in both the Shufeiya Recipe moderate- and high-dose groups were significantly elevated (*P* < 0.05, *P* < 0.01). The most marked improvement was obtained by the high-dose treatment of Shufeiya Recipe (Figure 4).

### 3.5. Effects on the Expression of NO, sGC, cGMP, and PKG

**Figure 5:**
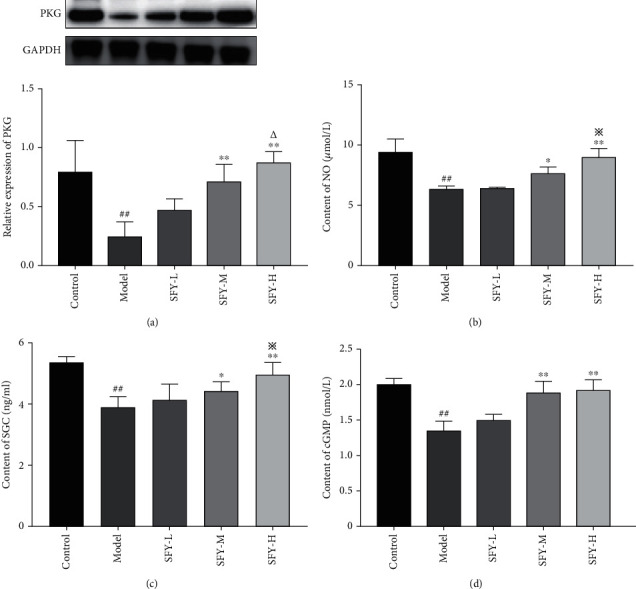
Shufeiya Recipe upregulated concentration of NO, sGC, and cGMP and the protein expression level of PKG in PH rats. (a) Moderate and high groups of Shufeiya Recipe upregulated the protein expression level of PKG, *n* = 3. (b) Moderate and high groups of Shufeiya Recipe upregulated the concentration of NO, *n* = 3. (c, d) Moderate and high groups of Shufeiya Recipe upregulated the concentration of sGC and cGMP, *n* = 6. ^##^*P* < 0.01 vs. the control group; ^∗^*P* < 0.05, ^∗∗^*P* < 0.01 vs. the model group; ^△^*P* < 0.05 vs. the low-dose group of Shufeiya Recipe; and ^※^*P* < 0.05 vs. the moderate-dose group of Shufeiya Recipe.

In contrast with the control group, the concentration of NO, sGC, and cGMP and the protein expression level of PKG in the model rats were considerably reduced (*P* < 0.01). Compared to the model group, moderate and high doses of Shufeiya Recipe could significantly upregulate the concentration of NO, sGC, and cGMP and the protein expression level of PKG (*P* < 0.05, *P* < 0.01) (Figure 5).

### 3.6. Effects on the Ras/ERK Signaling Pathway

**Figure 6:**
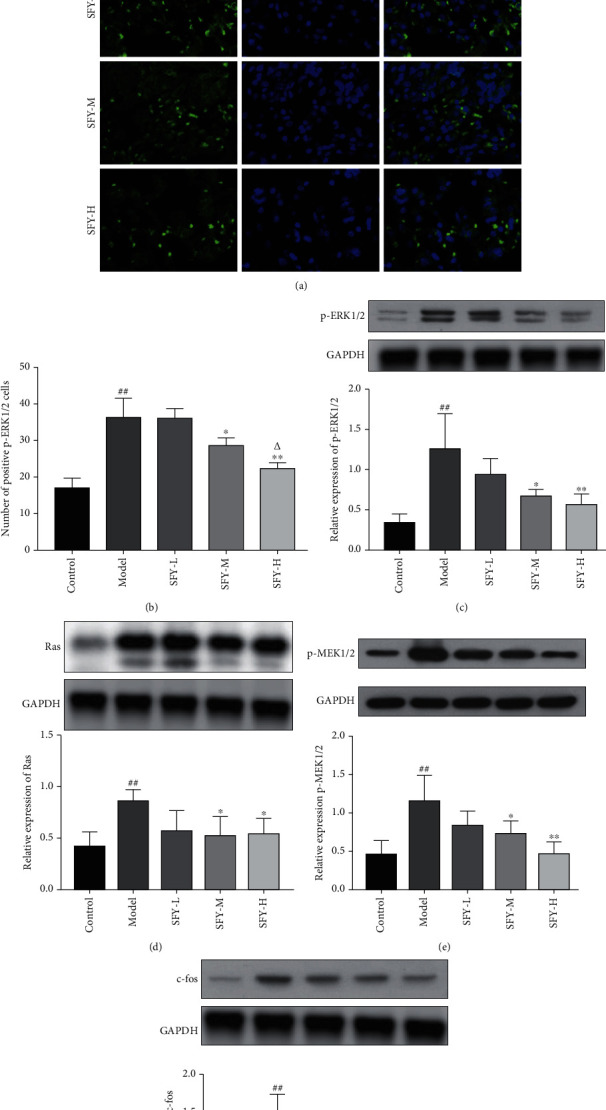
Shufeiya Recipe inhibited the Ras/ERK signaling pathway of PH rats. (a, b) Moderate and high groups of Shufeiya Recipe downregulated the relative immunofluorescence intensity of p-ERK1/2. (c–e) Different doses of Shufeiya Recipe downregulated the protein expression level of Ras, p-MEK1/2, p-ERK1/2, and c-fos. *n* = 3; ^##^*P* < 0.01 vs. the control group; ^∗^*P* < 0.05, ^∗∗^*P* < 0.01 vs. the model group; ^△^*P* < 0.05 vs. the moderate-dose group of Shufeiya Recipe.

The protein expression levels of Ras, p-MEK1/2, p-ERK1/2, and c-fos in the model rats were dramatically higher than those in the control group (*P* < 0.01). However, in contrast to the model group, administering moderate and high doses of Shufeiya Recipe could induce an obvious reduction in the protein expression of Ras, p-MEK1/2, and p-ERK1/2 in PH rats (*P* < 0.05, *P* < 0.01). Interestingly, only the Shufeiya Recipe high-dose group showed decreased c-fos protein expression level in PH rats (*P* < 0.01) (Figure 6).

## 4. Discussion

The development and progression of pulmonary hypertension are closely associated with the abnormal function and/or structure of pulmonary arterioles and pulmonary vessels. If not controlled well, it will eventually lead to right ventricular failure and even death in severe cases [29]. Pulmonary hypertension is preliminarily caused by long-term hypoxia or other factors that damage the structure and function of endothelial cells, causing imbalances of vasoactive substances such as NO and dysfunction of ion channels, resulting in abnormal endothelial cell barrier function and imbalance of secreted active factors, eventually leading to abnormal pulmonary vasoconstriction [30–32]. The pathological manifestations of PH are characterized by pulmonary artery intima injury and proliferation, hypertrophy and continuous contraction of the middle membrane layer, adventitia fibrosis, extracellular matrix remodeling, and inflammatory infiltration, to name a few. Besides, vasodilatation and in situ thrombosis can often be seen at the distal end of the lesion, leading to progressive stenosis and occlusion of the pulmonary artery lumen.

Oxidative stress is a key precursor to pulmonary hypertension. The imbalance between the generation and removal of oxygen free radicals leads to excessive ROS accumulation. ROS is directly related to pathological changes such as pulmonary artery smooth muscle cell proliferation, endothelial dysfunction, in situ thrombosis, inflammation, and vascular remodeling [33]. As a rate-limiting enzyme in the process of prostaglandin synthesis, COX-2 and its derivative metabolites have antioxidant properties [34]. It has been shown that knocking out the mouse COX-2 gene could increase the level of oxidative stress and the proliferation level of smooth muscle cells in mice with pulmonary hypertension [35]. COX-1 is a membrane penetrating protein complex, and changes in its level could cause a large amount of ROS to be released, while excessive ROS could affect the unsaturated fatty acids in the biofilm and cause lipid peroxidation [36].

As an important deacetylase in the mechanism of oxidative stress, SIRT3 has a regulatory effect on mitochondrial function. When mitochondrial dysfunction occurs, oxidative stress damage might follow [37]. Normally, cells can eliminate excess ROS through antioxidant enzymes such as Mn-SOD and CAT to maintain normal cell function. SIRT3 can not only directly regulate the activity of Mn-SOD and improve the function of ROS clearance [38] but also can deacetylate the forkhead transcription factor (FOXO3a), activate FOXO3a-dependent Mn-SOD and CAT, upregulate the expression of Mn-SOD, and inhibit excessive ROS generation. Excessive ROS can activate the Ras/ERK signaling pathway, and the activated Ras can cause the phosphorylation of its downstream substrate Raf kinase, upregulate the protein expression levels of its downstream substrates MEK1/2 and ERK1. Elk1 can, in turn, positively regulate the activity of the c-fos promoter, which induces the proliferation and differentiation of pulmonary artery smooth muscle cells and promotes the process of pulmonary hypertension [16].

In addition, the functional imbalance between ROS and the antioxidant system is one of the main causes of endothelial dysfunction [39]. Excessive ROS can inhibit the activation of the PI3K/AKT signaling pathway, resulting in a decrease in NO production. NO is produced by the enzymatic catalysis of endothelial nitric oxide synthase (eNOS), and previous studies in the pulmonary hypertension rat model have verified the involvement of NO in regulating eNOS phosphorylation and PI3K/AKT/eNOS and cGMP-PKG signaling pathways [40, 41]. Activated PI3K can phosphorylate lipids on the cell membrane and activate AKT, which can mediate downstream signals [42], which in turn causes conformational changes in the phosphorylation sites of Ser1177 and Thr495 of the eNOS amino acid residues to increase the activity of eNOS activity to promote the production of NO. Meanwhile, NO could activate sGC in vascular smooth muscle cells, and the activated sGC could catalyze the conversion of GTP into cGMP to activate cGMP-dependent protein kinase (PKG) [43], leading to vasodilation, thereby improving pulmonary hypertension.

Because traditional Chinese medicine has the advantages of being a multicomponent, multitarget, and multimechanistic treatment strategy, it has gradually received much attention in pulmonary hypertension therapy. Herein, we showed that Shufeiya Recipe, an empirical recipe for clinical treatment of pulmonary hypertension, has a good therapeutic potential. The *Radix Salviae* in Shufeiya Recipe mainly includes diterpenoids and phenolic acids, such as danshenol A, salvianolic acid A, and tanshinone IIA. These compounds have good pharmacological activities; for instance, the inhibition rate of the secretion of TNF-*α*, IL-1*β,* and IL-8 by danshenol A are 56.3%, 67.6%, and 51.7%, respectively [44]. Oxidative stress is an important mechanism in the occurrence and development of pulmonary hypertension, and salvianolic acid A and salvianolic acid B are famous for their strong antioxidant activities [45]. In addition, tanshinone IIA has been proved to inhibit myocardial remodeling, myocardial ischemia-reperfusion injury, and ischemic stroke and prevent liver cirrhosis by maintaining its antioxidant effect [46–48]. Studies have shown that tanshinone IIA has the pharmacological effects of activating the PI3K/AKT/mTOR signaling pathway, reducing oxidative stress, reducing expression of high-mobility group box B1 protein (HMGB1), and inhibiting inflammation [49–51]. In addition, Danshensu has shown a cardioprotective effect on isolated rat hearts by activating the AKT/ERK1/2/Nrf2 signaling pathway [52].

**Figure 7:**
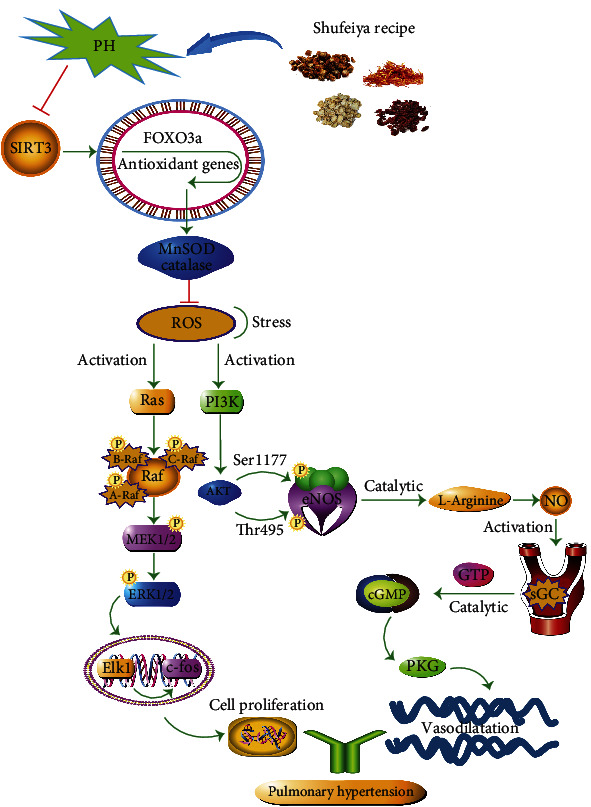
The protective mechanism of Shufeiya Recipe regulated PH

In addition, the main ingredients of *Carthami Flos* in the Shufeiya Recipe include safflower yellow and hydroxysafflor yellow A, which are often used to treat cardiovascular and cerebrovascular diseases [53]. They could also upregulate SOD activity and inhibit malondialdehyde (MDA) and ROS accumulation [54–56]. In particular, the flavonoids in *Carthami Flos* have a good regulatory effect on oxidative stress response [57] and could inhibit NO production and inflammatory response by decreasing the protein expression of iNOS and COX-2 genes [58]. *Cornus Officinalis Sieb. Et Zucc.* can increase the activities of SOD, CAT, and GPX [59], and the crude extract of *Cornus Officinalis Sieb. Et Zucc.* can regulate the protein expression levels of PI3K, Ras, and p-ERK1/2 [60]. Morroniside is an important active ingredient of *Cornus officinalis Sieb. Et Zucc.*, which can inhibit the expression of iNOS and COX-2 by regulating the NF-*κ*B signaling pathway [61]. *Platycodon Grandiforus* mainly contains triterpenoid saponins and platycosides, which can suppress chronic airway inflammation and oxidative stress damage by regulating the ROS/ERK signaling pathway [62] as well as reducing the thickness of alveolar septa [63]. Network pharmacology studies have shown that *Platycodon Grandiforus* could also improve a variety of pathological changes in the heart and lungs by regulating the PI3K/AKT signaling pathway [64]. These above studies provide a theoretical basis for Shufeiya Recipe in treating pulmonary hypertension. This study drew a conclusion that Shufeiya Recipe could differentially downregulate mPAP in PH rats and improve lung histopathological changes. Besides, it could downregulate ROS levels, upregulate the concentrations of COX-1 and COX-2 and the activity of Mn-SOD, and inhibit oxidative stress damage. Additionally, it could upregulate the protein expression levels of SIRT3 and FOXO3a; promote the protein expressions of p-PI3K, p-AKT, and p-eNOS; and increase the expression levels of NO, sGC, cGMP, and PKG. Moreover, it could downregulate the protein expression levels of Ras, p-MEK1/2, p-ERK1/2, and c-fos (Figure 7).

## 5. Conclusion

In summary, our results indicate that Shufeiya Recipe can improve MCT-induced pulmonary hypertension in rats by regulating SIRT3/FOXO3a and its downstream PI3K/AKT/eNOS and Ras/ERK signaling pathways.

## Data Availability

The data used and/or analyzed during the current study are available upon direct request to the corresponding author.
